# Dissecting the Heterogeneity of Circulating Tumor Cells in Metastatic Breast Cancer: Going Far Beyond the Needle in the Haystack

**DOI:** 10.3390/ijms17101775

**Published:** 2016-10-24

**Authors:** Michela Bulfoni, Matteo Turetta, Fabio Del Ben, Carla Di Loreto, Antonio Paolo Beltrami, Daniela Cesselli

**Affiliations:** 1 Department of Medical and Biological Sciences, University of Udine, Piazzale M. Kolbe 4, 33100 Udine, Italy; michela.bulfoni@alice.it (M.B.); matteosalotto@gmail.com (M.T.); carla.diloreto@uniud.it (C.D.L.); antonio.beltrami@uniud.it (A.P.B.); 2 Department of Clinical Pathology, CRO Aviano National Cancer Institute, via F. Gallini 2, 33081 Aviano, Italy; fdbf13@gmail.com; 3 Institute of Pathology, University Hospital of Udine-ASUIUD, Piazzale Santa Maria della Misericordia 15, 33100 Udine, Italy

**Keywords:** circulating tumor cells, spatial and temporal heterogeneity, metastatic breast cancer, epithelial-to-mesenchymal transition, metabolism, stemness

## Abstract

Although the enumeration of circulating tumor cells (CTC) defined as expressing both epithelial cell adhesion molecule and cytokeratins (EpCAM^+^/CK^+^) can predict prognosis and response to therapy in metastatic breast, colon and prostate cancer, its clinical utility (i.e., the ability to improve patient outcome by guiding therapy) has not yet been proven in clinical trials. Therefore, scientists are now focusing on the molecular characterization of CTC as a way to explore its possible use as a “surrogate” of tumor tissues to non-invasively assess the genomic landscape of the cancer and its evolution during treatment. Additionally, evidences confirm the existence of CTC in epithelial-to-mesenchymal transition (EMT) characterized by a variable loss of epithelial markers. Since the EMT process can originate cells with enhanced invasiveness, stemness and drug-resistance, the enumeration and characterization of this population, perhaps the one truly responsible of tumor recurrence and progression, could be more clinically useful. For these reasons, several devices able to capture CTC independently from the expression of epithelial markers have been developed. In this review, we will describe the types of heterogeneity so far identified and the key role played by the epithelial-to-mesenchymal transition in driving CTC heterogeneity. The clinical relevance of detecting CTC-heterogeneity will be discussed as well.

## 1. Introduction

An effective anticancer treatment must be able to eradicate all tumor cells; additionally, recent therapeutic advances in oncology recognize a patient-to-patient variability in the mutational status of tumors, the so-called interpatient heterogeneity. This latter dictates the choice of targeted drugs [1]. However, tumors are evolving entities and genetic heterogeneity has been detected analyzing different regions of the same tumor (i.e., “spatial heterogeneity”), or comparing the primary tumor with subsequent recurrences and metastases (i.e., “temporal heterogeneity”) [1]. Therefore, it has been hypothesized that the success of personalized treatments greatly depends on the capability to capture and monitor tumor heterogeneity over time and to consequently modulate therapies [2].

However, although nowadays sequencing technologies make in principle feasible the identification of genetic variants within a biological sample, it is a challenge to characterize intratumor heterogeneity at diagnosis as well as to monitor clonal dynamics during treatment, promptly detecting the appearance of resistant clones. In fact, the analysis of a single bioptic or surgical sample cannot detect all the genetic variants present in a tumor [1,3], while obtaining multiple and serial tissue samples during disease progression is often difficult for practical reasons [4].

Therefore, great interest is now focused on the opportunity to monitor tumor genome evolution by the characterization of cell-free circulating tumor DNA, circulating tumor-derived exosomes and circulating tumor cells (CTC) [4]. This approach, known as “liquid biopsy”, requires a simple blood draw, a minimally invasive practice that can be easily repeated over time, thus ensuring a constantly open window on the genomic landscape of the tumor [5]. However, despite the ease of the sampling procedure, cell-free circulating tumor DNA, exosomes and CTC are extremely rare events dispersed in the blood and their identification, quantification and analyses have requested and favored the development of multiple new technologies.

In this review, we will focus on CTC, with a particular interest in the metastatic breast cancer (MBC) scenario, trying to define: (1) the assumptions that led to consider significant not only the enumeration but also the characterization of CTC; (2) the types of heterogeneity so far identified; and (3) the role played by the epithelial-to-mesenchymal transition in CTC heterogeneity. The clinical relevance of detecting CTC-heterogeneity will be discussed as well.

## 2. CTC: From Quantity to Quality

### 2.1. CTC: The Premise

CTC are rare cells released into the blood stream by primary or metastatic tumors [6,7]. CTC have been identified in many solid tumors including breast [8], prostate [9], lung [10], bladder [11], gastric [12], and colon cancer [13], while they are rarely found in healthy people or in people with nonmalignant tumors [10].

Once in the circulation, CTC evade immune detection and could extravasate into microvessels of target tissues such as lymph nodes, bones, liver, brain, and lungs [14,15,16]. In fact, the successful formation of a metastatic lesion seems to be dependent upon the CTC’s ability to adapt, survive, and induce neoangiogenesis in the target tissue [17]. Consistently, in the blood of MBC patients the existence of CTC able to initiate metastasis in xenograft assays was demonstrated [6,7]. Besides, it has been shown that CTC can colonize their tumors of origin too, in a process called “tumor self-seeding”, promoting tumor growth, angiogenesis, and stromal recruitment through seed-derived factors [18]. Importantly, it has been demonstrated both in a genetic model of pancreatic ductal adenocarcinoma [19] and in some preneoplastic human conditions [20,21] that CTC can be detected in the bloodstream before tumor formation, supporting the notion that CTC release can be an early event during tumorigenesis.

However, studies on CTC have been conducted not only to get insights into the metastatic process, but also to assess their clinical utility as surrogate biomarkers in the management of cancer patients [5,22]. In this regard, the objectives of the research on CTC comprise: (a) assessment of patient prognosis; (b) prediction and monitoring, in real-time, of the response to treatments; and (c) identification of new drugs and pathways responsible for drug resistance [5].

### 2.2. CTC as Surrogate Biomarkers

Since the beginning, two levels of CTC analyses have been postulated: CTC enumeration and CTC characterization [23]. CTC enumeration could serve to prognostically stratify patients, as an early marker of response to systemic therapy, to detect disease in patients at high-risk of developing cancer and monitor cancer recurrence. Conversely, CTC molecular characterization could be instrumental to assess tumor heterogeneity, to predict site-specific metastases, to detect treatment-resistant profiles and to identify new drug targets [23]. The possibility of using CTC as a “surrogate” of the tumor tissue to monitor in real time changes in the genomic landscape of the neoplasia can favor a personalized therapeutic approach aimed at sparing patients useless and potentially toxic treatments [22].

However, both robust CTC methodologies and well-designed clinical trials are requested to confirm the clinical utility of CTC detection and analyses in tumor patients. As we will describe below, plenty of promising methods have been developed in recent years to detect, quantify and characterize CTC. Unfortunately, only for a few of them a clinical validation (i.e., the association with specific clinical outcomes) is available (Table 1), while no one has yet demonstrated a clinical utility (i.e., the ability to improve the patient survival by guiding the clinical management). Specifically, the CellSearch™ system is the only technology that has received the approval by FDA for the quantification of CTC in specific clinical settings [24]. In metastatic breast, prostate and colorectal cancer, a number of CTC higher than a specific cut-off level is associated with a poor prognosis, while changes in the number of the CTC upon the commencement of a new therapy are endowed with both a prognostic and a predictive value [8,25,26]. Specifically, in MBC patients, a number of CTC higher than 5 in 7.5 mL of blood at baseline was associated with a reduced progression-free survival (PFS) and overall survival (OS). Moreover, at the follow-up after the beginning of a new therapy, the conversion from an elevated CTC number to a low one was associated with an improved prognosis.

The success of the CellSearch™ platform is due to the fact that this semi-automated system has proven to be reproducible, reliable, sensitive, linear and accurate [10,27], thus allowing clinical testing across multiple centers. Consistently, in 2012, a meta-analysis evaluating 49 eligible studies enrolling 6825 patients demonstrated that the detection of CTC was a stable prognosticator in patients with early-stage and metastatic breast cancers, independently from the method used and the time of analysis [28]. Similarly, two European multicenter studies, based on a pooled analysis of individual patient data, confirmed the independent prognostic effect of CTC count, as assessed by the CellSearch™ system, on progression-free survival (PFS) and overall survival (OS) of both metastatic [29] and non-metastatic breast cancer patients [30].

However, to demonstrate the clinical utility of CTC, well-designed interventional clinical trials are requested; that is, prospective, randomized, multicenter clinical trials showing that a change in therapy based on CTC evaluation significantly impacts patient outcomes. In this regard, a prospective, randomized clinical trial, SWOG S0500 (NCT00382018), was designed to assess whether MBC patients that, after one cycle of first-line chemotherapy still present a high CTC number, could benefit of an alternative drug treatment [31]. Although the study confirmed the prognostic significance of CTC in MBC patients, the rapid change of therapy did not improve OS and PFS of patients with persistently increased CTC after three weeks of therapy [31]. According to authors, the disappointing lack of improved outcome in early-switching patients does not question the potential clinical utility of CTC; rather, it suggests that, in MBC patients, the failure of a first line chemotherapeutic regimen to reduce CTC after one cycle might indicate a general chemotherapy resistance [31]. In this case, molecular analyses of CTC might determine whether such patients are eligible for trials based on targeted therapy. Accordingly, the focus has been recently shifted from the simple enumeration to the assessment of specific CTC phenotypes and interventional trials based on this principle are ongoing [30], as we will discuss later. Nonetheless, other interventional trials based on CTC number are still ongoing. In the French CirCé01 trial (Circulating Tumor Cells to Guide Chemotherapy for Metastatic Breast Cancer; NCT01349842), patients will be switched to different chemotherapeutic drugs, depending on the (in)effectiveness of the chosen drug to reduce CTC counts. Patients will be assessed for drug resistance, by means of CTC enumeration, whenever they will start a new chemotherapy line, and this approach will be repeated until a drug able to produce a sufficient drop of the CTC count will be found [32]. The idea is to avoid patient useless and toxic therapies.

### 2.3. CTC: Open Questions10.3390/ijms17101775

As demonstrated by the SWOG S0500 trial, the simple enumeration of CTC is not sufficient to guide therapy. In this trial, CTC were measured by the only FDA-approved CTC detection method, the CellSearch system. However, this method presents some limitations. In fact, the CellSearch system enriches for CTC using an immunomagnetic bead-based separation strategy directed against the epithelial-specific marker EpCAM. One potential problem with the use of an epithelial-specific marker is that tumor cells undergoing epithelial-to-mesenchymal transition (EMT) are considered to be more invasive [55,56]. This process leads, in tumor cells, to a reduction in epithelial marker expression and an increase in mesenchymal proteins, which is paralleled by the loss of cell-cell adhesions and a subsequent increase in migration and invasive abilities as well as in stem cell properties [57,58,59]. Therefore, the CellSearch system might underestimate those CTC that are more invasive and endowed with the highest metastatic potential [60]. Indeed, EpCAM-negative CTC have been detected in the blood of patients with specific subtypes of MBC [61,62], such as the basal-like one [63]. Consistently, Marchetti’s group identified CTC with brain metastatic capabilities as EpCAM-negative [7]. Moreover, even in cells positive for epithelial markers, a variable expression of both mesenchymal and stem cell markers was documented [57,59]. Yu’s group showed that the expression of epithelial and mesenchymal markers presents dynamic changes during MBC progression [50].

The necessity to better investigate CTC at single cell level, possibly extending analyses to the genetic and transcriptional landscape, as well as to functional properties, has revealed two other limitations of the CellSearch system, namely the fact that it simply analyzes, but it does not sort fixed, and therefore not viable, cells. Thus, in an attempt to overcome these limitations, innumerable groups have developed new devices able not only to isolate CTC independently from the EpCAM expression, but also to isolate and sort single, possibly viable CTC for downstream molecular and functional analyses [64,65].

Methods for CTC detection employ a vast array of strategies, which include selection on biophysical or metabolic properties as well as on more “specific” biological features, such as tumor cell surface marker expression. For an updated and careful review of the different CTC technologies see [64]. From this list of technologies it is clear that there is no single definition of CTC and no single CTC biomarker [66]. There are, instead, multiple assays (tests) to detect CTC [66]. In an attempt to evaluate and compare these different technologies, a standard set of performance criteria has been defined: capture efficiency, purity, enrichment, throughput, cell viability, and release efficiency [64]. However, these parameters were assessed using spiked sample, i.e. blood samples added with a known number of tumor cells deriving from commercially available cell lines. These latter are not able to recapitulate the physical and biological heterogeneity of patient-derived CTC. Therefore, while, on the one hand, by using spiked samples, the device performance is over predicted, on the other hand, patient-derived samples cannot be directly employed to validate novel devices, since a reference method to measure CTC does not exist. Nonetheless, CellSearch is often used as the reference technique for the enumeration of epithelial CTC.

Therefore, it is difficult to compare cell populations isolated by adopting different strategies and this method-dependent heterogeneity can be added to the intrinsic heterogeneity of CTC. For this reason, it is considered to be essential, for each novel CTC test: (1) to define the context of use (i.e., diagnostic, prognostic, predictive, or surrogate of efficacy of response); (2) to obtain an analytical validation (i.e., assessing the assay and its measurement of performance); (3) to obtain a clinical validation (i.e., evaluating the strength of the association with specific clinical outcomes); and (4) to establish its clinical utility (i.e., the capacity to impact patient prognosis by guiding the clinical management) [66].

## 3. CTC Intrinsic Heterogeneity

As explained before, the interest in CTC heterogeneity derives from the intent to obtain information that is useful to direct the therapeutic strategy with a consequent improvement in the patient’s prognosis. Thanks to the large number of strategies that have been used to identify and characterize CTC, several forms of heterogeneity have been described and investigated. The underlying theme has been to identify specific sub-populations whose targeting can greatly contribute to the disease control. As described below, in some cases the heterogeneity has been only described, in others some clinical proofs of the prognostic/predictive significance of specific features/subpopulations have been reached (Table 1), while in very few cases the evidences reached were so strong to initiate interventional clinical trials, still ongoing, to definitely assess the clinical utility of CTC characterization.

### 3.1. Biophysical Features: Small Size CTC and Clusters

Although size has been used to separate CTC from the smaller blood cells, both “small CTC” [67,68] and clusters [50,69,70,71] have been described.

The first ones have been mainly described in patients with prostate cancer. Specifically, CTC were captured and enumerated on NanoVelcro Chips and subsequently sub classified on the basis of nuclear size. Authors revealed the existence of very small nuclear CTC whose detection was correlated with the presence of visceral metastases [67]. Similarly, Attard’s group using the Epic CTC Platform showed the presence of small CTC in patients with metastatic castration-resistant prostate cancer [68].

CTC-clusters, of various tumor origin, were identified employing different methodologies [50,69,71,72,73]. Clusters are formed by cells often co-expressing epithelial and mesenchymal markers, indicating a hybrid or partial EMT [50,70]. This latter status, not only can favor cluster formation, but is associated with drug resistance and tumor-initiating potential [70]. Consistently, Aceto’s group employed elegant animal models to demonstrate the oligoclonal origin of CTC-clusters. Importantly, with respect to single CTC, circulating clusters were characterized by a higher metastatic potential [69]. Importantly, the presence of CTC-clusters, as assessed by Herringbone-Chip or CTC-iChip, could predict a poor prognosis, in patients affected by prostate and breast cancer. Interestingly, a primary role in mediating tumor cell clustering was played by plakoglobin [69], thus offering an opportunity for novel therapeutic interventions [69]. These results have induced Toner’s group to develop a microfluidic device specifically dedicated to detect CTC-clusters [73].

### 3.2. Identification of CTC Subsets with Specific Properties

The number of CTC, at least as identified by CellSearch (EpCAM^+^CK^+^DAPI^+^CD45^−^), has a prognostic/predictive value, but is unable, per se, to guide therapy, thus impacting the patient outcome [31]. As previously described, novel devices and strategies have been optimized to detect those CTC that are possibly lost by CellSearch, thus improving the “CTC count” ability. Simultaneously, several groups have focused their attention on identifying, within the CTC pool, subsets of cells endowed with stem cell- and metastasis initiating-potential. Since these cells are perhaps the truly responsible for tumor recurrence and progression, their enumeration and characterization can become more clinically useful.

#### 3.2.1. CTC in Epithelial-to-Mesenchymal Transition

The process of epithelial-to-mesenchymal transition (EMT) leads to the loss of epithelial markers such as E-cadherin and EpCAM, over expression of N-cadherin, and cytoskeletal alterations (e.g., expression of vimentin), finally producing phenotypical and structural changes that lead to an increased motility and invasiveness [38,57,74,75]. Importantly, intermediate phenotypes between epithelial and mesenchymal differentiation can exist. Therefore, CTC studies have tried to address the presence of CTC in EMT, to define their phenotype and to establish their clinical relevance.

In this regard, the first studies were mainly focused on the demonstration of mesenchymal transcripts in samples enriched in epithelial CTC by AdnaTest [42,55,76] or by immunomagnetic selection of EpCAM-positive cells [54,59]. Aktas et al. showed that in MBC patients, EMT markers were detected in more than half of the blood samples containing CTC. Moreover, patients resistant to therapy were more frequently positive to EMT markers [42]. Interestingly, a certain fraction of samples negative for CTC expressed EMT markers. Kasimir-Bauer et al. obtained similar results in primary breast cancer patients [76]. Again, Raimondi et al. showed that more than a third of CTC negative MBC patients expressed EMT markers [59]. Similarly, Giordano’s group showed, in HER2 MBC patients, the expression of EMT markers not only in CTC but also in the CD45^−^/EpCAM^−^ fraction of 60% of patients [54]. Mego’s group, adopting a slightly different protocol analyzing, in the CD45 negative fraction of MBC patients, both the presence of EpCAM-positive CTC by flow-cytometry and of mesenchymal transcripts by RT-PCR, showed a reduced progression free survival in patients with high levels of EMT transcripts [77].

All these results supported the existence of CTC in EMT and suggested that part of CTC in EMT could be lost by the common CTC-enrichment strategies relying on epithelial markers [78]. Nonetheless, other studies confirmed, by immunocytochemistry, the co-expression, in single CTC, of epithelial and mesenchymal markers [79,80]. In this regard, Kallergi showed that CTC expressing Twist and vimentin were increased in patients with a metastatic disease, with respect to those with an early-stage breast cancer, thus supporting the hypothesis that the EMT process could be involved in the metastatic cascade [81]. More recently, Yu’s group, as previously mentioned, analyzing by FISH the expression of epithelial and mesenchymal transcripts in CTC enriched by EpCAM and HER2, showed a variable expression of these markers in CTC [50]. Interestingly, while luminal-type MBC patients were mainly characterized by epithelial CTC, triple negative- and HER2-derived CTC expressed mostly mesenchymal transcripts [50]. These data confirmed other reports showing an increased fraction of CTC in EMT in patients with HER-2 MBC [54]. Moreover, in an index patient, mesenchymal CTC resulted to increase during cancer recurrence, while epithelial CTC were prevalent during the phase of response to therapy [50]. This implies the possible usefulness of a real time monitoring of the changes in CTC number and phenotype during the patient follow-up. Possibly, sorting cells at different time points and associating the phenotype with genetic features could add further information for the comprehension of the disease evolution.

**Figure 1:**
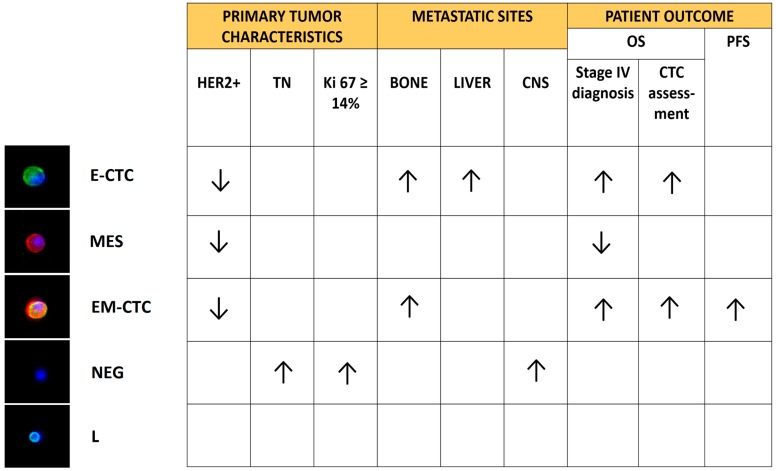
CTC in EMT can predict prognosis. CD45-depleted blood samples of MBC patients were assessed for the presence of nucleated cells (recognized by the blue staining of DAPI) expressing epithelial (green fluorescence), mesenchymal (red fluorescence) and leukocyte (cyan fluorescence) markers. As depicted on the left panels, besides CD45-positive leukocytes (L), 4 subsets of CD45-negative cells were detected: cells expressing only epithelial markers (E-CTC), cells co-expressing epithelial and mesenchymal markers (EM-CTC), cells expressing only mesenchymal markers (MES) and cells negative for all the assessed markers (NEG). Increased (↑) or decreased (↓) number or proportion of these subsets was significantly associated with specific clinical-pathological features and patient outcome. OS, overall survival; PFS, progression free survival; TN, triple negative; CNS, central nervous system [46].

To evaluate whether CTC in EMT were associated with both the clinicopathological characteristics and outcome of 56 MBC patients, we optimized a DEPArray-based methodology to enumerate, sort and characterize single, viable CTC in EMT (Figure 1) [46]. In order to avoid the loss of EpCAM-negative CTC, CD45 expressing cells were removed from blood samples, employing magnetically labeled antibodies. The remaining cells were labeled with antibodies against epithelial (EpCAM and E-cadherin), mesenchymal (CD44, CD146 and N-cadherin) and leukocyte (CD45) markers. The CD45-negative fraction was constituted of four cell subsets: epithelial CTC (E-CTC), CTC in EMT (EM-CTC), mesenchymal cells (MES) and cells negative for every tested marker (NEG). This prospective observational study demonstrated, after a quantification of the different CD45 negative subpopulations present in every blood sample, that specific subpopulations were associated with: tumor subtypes (e.g., NEG and triple negative tumors), proliferation index of the primary tumor (e.g., NEG and high Ki67 expression) and metastatic sites (e.g., E-CTC and bone; NEG and brain) (Figure 1). Moreover, high EM-CTC counts were predictive of poor PFS and OS. This latter was evaluated both from the initial CTC assessment and from the diagnosis of a metastatic disease [46]. These two ways of computing OS are conceptually related to two different models of cancer pathophysiology. The first one hypothesizes that cancer is characterized by a spatial- and temporal-heterogeneity and CTC analyses can be employed to monitor, in real time, the metastatic dissemination [38]. On the contrary, according to the second one, the detection of EM-CTC seems to predict a metastatic disease that is intrinsically drug-resistant. This is interesting in view of the SWOG S0500 results, indicating how patients that did not reduce CTC number upon the first cycle of chemotherapy did not benefit of an early switch to another therapeutic protocol [31]. In any case, a comprehensive analysis of the genomic landscape of these four different CD45-negative populations could get insight into their nature and add information for the comprehension of the tumor evolution.

#### 3.2.2. CTC with Stem Cell Properties

Cancer stem cells represent a rare population of tumor cells that, because of their unique self-renewal and multilineage differentiation properties, not only are responsible for the tumor initiation and maintenance [82], but also are considered the source of the metastatic tumor spread [57]. Cancer therapies unable to kill cancer stem cells are inevitably destined to fail with a consequent progression of the tumor or the development of local and distant recurrences, even after years. Therefore, identifying and characterizing CTC with stem cell properties could help in predicting the risk of metastasis and in devising therapeutic strategies aimed at specifically targeting this population.

The identification of CTC with stem cell properties has been based on the recognition, on the surface of single cells, of known stem cell proteins, or on the quantification, on CTC-enriched samples, of stem cell related transcripts [57]. For example, Theodoropoulos et al., using triple-marker immunofluorescence microscopy after density gradient centrifugation, could identify on CTC of MBC patients the expression of CD44, CD24 and Aldehyde Dehydrogenase 1 (ALDH1), markers known to be associated with stemness and enhanced tumorigenic potential in breast cancer [83]. Using flow-cytometry, Giordano et al. could detect in HER2-MBC patients a subpopulation of cancer stem cells expressing ALDH1, CD44, and low amounts of CD24 or ALDH1 and CD133 [54]. These populations seemed to be higher in those patients characterized by an increased number of CD45^−^EpCAM^−^ cells. The transcript of ALDH1 has been also evaluated by RT-PCR in CTC enriched by AdnaTest or flow-sorter, sometimes in combination with other EMT markers or stem cell-related markers, such as Bmi1, CD133 and CD44 [42,55,57,76,77].

Thus far, it has been demonstrated that: (1) CTC with some stem cell features can be detected in the blood of breast cancer patients affected by either a metastatic disease or by a primary tumor only [42,54,55,57,76,77,83]; (2) CTC of breast cancer patients frequently coexpress stem cell and EMT markers, supporting the notion that EMT is associated with the acquisition of stem cell-like features (see below) [84,85]; and (3) Consistent with the notion that EpCAM could be lost by CTC in EMT, the presence of stem cell markers has been also detected in samples considered to be negative for EpCAM-positive CTC [42,54,76].

Regarding the clinical consequences, Raimondi showed that an over expression of stem cell markers in CTC correlated to the stage of disease [59]. Moreover, the presence of stem cell-like CTC in peripheral blood of MBC patients was associated with therapy resistance; specifically, 74% of patients non-responding to systemic treatment were characterized by the expression of EMT and stem cell markers [42]. Accordingly, drugs targeting the pathways responsible for both EMT and stemness are now under evaluation at pre-clinical and clinical level [57]. This mechanism of action has been hypothesized to be responsible for the ability of everolimus, an inhibitor of the PIK3/Akt/mTOR pathway, to effectively target breast cancer cells resistant to standard therapy [86]. Consistently, the ability of everolimus to restore sensitivity to the following drugs has been/is evaluated in clinical trials: tamoxifene (phase II study) [87]; nonsteroidal aromatase inhibitor (phase III BOLERO-2 trial) [88]; and trastuzumab (phase III BOLERO-1 and phase III BOLERO-3 trial) [89,90]. Similarly, inhibitors of other signaling pathways crucial for the survival and self-renewal of breast cancer stem cells are now under investigation in phase I and phase II clinical trials [57,91].

Recently, it has been shown that HER2 was selectively expressed in and regulated self-renewal of the cancer stem cell (CSC) population of luminal estrogen receptor-positive and HER2-negative breast cancers [92]. This can explain the clinical efficacy of adjuvant trastuzumab in HER-2 negative tumors, where this agent could target the CSC population in a process that does not require HER2 gene amplification [92]. Consistently, it has been demonstrated, in an in vitro study, that IL6 was instrumental for the development of trastuzumab resistance by promoting CSC proliferation. Blocking this inflammatory cytokine could provide an alternative strategy to overcome trastuzumab resistance [93].

Altogether, these findings support the idea that a maximal clinical benefit could be achieved when CSC-targeting agents are administered. In this regard, molecular studies specifically directed on CTC with stem cell feature can suggest additional druggable targets.

#### 3.2.3. CTC Undergoing Metabolic Reprogramming

The metabolic reprogramming is considered to be one of the hallmarks of cancer [94]. Specifically, cancer cell metabolism largely relies on glycolysis, independently from oxygen availability. As a result, large amounts of lactate are produced and released, leading to an acidification of the tumor environment. This property has been named “Warburg effect” or “aerobic glycolysis” [95]. For this reason, a metabolic-based approach to detect CTC has been developed from our group [96]. Since CTC are extremely rare, they cannot modify the pH or lactate levels of large volumes of blood. Therefore, it has been optimized a device that: (1) employs microfluidic technologies to compartmentalize blood cells in microfluidically prepared, monodisperse, pL droplets, each containing single cells; and (2) detects and quantifies in real time the production of lactate or hydrogen ions by single cells, by employing a setup that utilizes an inverted microscope to interrogate droplets that flow into a microfluidic channel, by a laser-induced fluorescence [96]. Fluctuations in pH or lactate concentration can be employed to identify putative CTC without the need for surface-antigen labeling [96]. The device was effective in specifically recognizing the presence of both tumor cells of different cell lines, in spiked samples, and putative CTC, in the blood of metastatic patients. Although further work is needed to confirm these results and clarify their clinical value [96], this method is an easy and inexpensive approach to detect and sort single viable CTC that are not required to express specific antigens and could be suitable for downstream analyses. Moreover, it has the potential to be employed to test novel therapeutic strategies, exploiting, in ex vivo functional assays, the synergistic effects of new drugs with conventional anti-cancer agents.

#### 3.2.4. CTC with Metastasis-Initiating Properties

Very few works have grappled with the identification of CTC subpopulations capable of initiate metastases when injected into immunodeficient animals [65]. This has required the development of protocols to sort viable CTC starting from large blood volumes (being CTC extremely rare), as well as the optimization of long-term culture protocols and of patient-derived xenografts [65].

In 2013, Marchetti’s group established long-term primary cultures of CTC isolated from the blood of MBC patients with brain lesions [7]. It identified that CTC negative for EpCAM, co-expressing HER2, EGFR, Heparanase, and Notch1, showed a very high metastatic potential, with a special tropism for the lungs and the brain [7]. Bacelli’s group prospectively isolated, from luminal breast cancer patients in advanced metastatic state, CTC that metastasized to bone, lung and liver of xenografted immunodeficient mice [6]. Specifically, CTC enriched in metastasis-initiating cells were positive for EpCAM, CD44, CD47 and MET. These markers were also uniformly expressed by developed metastases, suggesting their possible role in the engraftment and metastatic potential of CTC [6]. More recently, Zamarchi’s group confirmed the engraftment capacity of EpCAM positive CTC when injected subcutaneously into NOD/Scid mice [97].

Altogether these reports suggest that specific subsets of CTC might have a metastasis-initiating activity and that these subsets can be phenotypically different depending on the metastatic site. Accordingly, we identified, in MBC patients, an association between specific CTC subpopulation and distinct metastatic sites [46]. Although promising, these pioneering works require to be further validated, possibly in studies involving not only patients with advanced metastatic cancer but also with an early-stage disease. Nonetheless, the possibility to identify CTC able to initiate metastases in different organs opens the way to further strategies aimed at using this information for diagnostic, prognostic and therapeutic purposes.

#### 3.2.5. Apoptotic CTC

In 2001, Mehes’s group showed that in MBC patients most of the CTC are indeed apoptotic, as assessed by morphological criteria and by the presence of apoptosis- related DNA strand breaks [98], supporting the notion that metastatization is an extremely inefficient process [74]. Rossi’s group confirmed these results in 2010. It took advantage of the CellSearch technology, implementing the platform with the evaluation of the presence of the M30 neoepitope, to enumerate apoptotic CTC in epithelial tumors [36]. M30 is a neoepitope disclosed by caspase cleavage at cytokeratin 18 in early apoptosis [36]. Depending on the histotype, 50% to 80% of CTC resulted to be apoptotic in patients not exposed to chemo- or radio-therapy. Even more interestingly, in a small case series of breast cancer patients, changes in the proportion of M30-expressing CTC during treatment could be used to evaluate in real-time drug response/resistance [36]. The role of dynamic changes of live/apoptotic CTC as predictive marker of response to sunitinib was confirmed in another clinical setting, the metastatic renal cancer [99]. Similarly, Kallergi’s group, studying the expression of both M30 and Ki67 in CTC of breast cancer patients demonstrated that apoptotic CTC could be detected in patients with either primary or metastatic breast cancer, though the incidence of detection was lower in metastatic patients [81]. Upon adjuvant therapy, there was only a decrease in the fraction of apoptotic CTC. This suggests that detection, enumeration and characterization of CTC that survive despite adjuvant treatment can be used to devise therapeutic strategies aimed at targeting this resistant population [81].

#### 3.2.6. Drug-Targetable CTC

There are increasing evidences that cancer evolves over time as a consequence of its genomic instability and under the selection pressure of systemic treatments. CTC analyses, providing a repeatable and minimally invasive approach, could allow a real-time monitoring of changes in the genomic landscape of the tumor. These changes can be responsible for the appearance of drug-resistant clones, which can nevertheless benefit from alternative therapeutic strategies. Therefore, analyses of CTC could provide relevant information for personalized therapies.

With regard to MBC, a discrepancy was observed between metastases or CTC and the primary tumors in terms of human epidermal growth factor receptor 2 (HER2), estrogen and progesterone (ER and PgR) receptor expression [30,100]. Specifically, analyzing HER2 status in CTC, either by immunofluorescence or fluorescence in-situ hybridization (FISH), authors showed the presence of HER2-positive CTC in patients with primary tumors negative for HER2 [37,40,49,101]. Consistently with the clinical relevance of this occurrence, interventional trials, still ongoing, have been designed. Specifically, DETECT III is a multicenter, randomized, phase III study aimed at evaluating the efficacy of treating HER2-negative MBC patients with anti-HER2 therapies whenever HER2-positive CTC are detected by CellSearch [30].

Interestingly, it has been shown that, in breast cancer patients, alterations of phosphatidylinositol 3-kinase (PIK3)-pathways can induce resistance to anti-HER2 therapies [102,103,104] and that mutations of the catalytic subunit of PIK3 (PIK3CA) can be present in the metastasis but not in the primary tumor [105]. This has suggested that the detection of these activating PIK3CA mutations in CTC could suggest a disease resistant to HER2 targeted therapies. Several groups have indeed shown the heterogeneity of PIK3CA gene status in single-sorted CTC analyzed at single cell level [47,48,106]. Accordingly, in DETECT III, a SNaPshot technology will be employed to assess the mutational status of PIK3CA in HER2 positive CTC [30,107].

Similarly, changes in the hormone receptor (ER and PgR) status have been demonstrated comparing CTC with primary tumors [108,109], and also CTC with metastases [100]. While the loss of hormone receptor expression in CTC was described in 40% of receptor-positive MBC, a gain in hormone receptor expression was detected in only 8% of triple negative MBC [100]. Intriguingly, comparing primary tumors, metastases and CTC regarding ER and PgR expression, primary tumors and metastases were concordant, while primary tumors and CTC, as well as metastases and CTC, were not [100]. In this regard, the translational research project DETECT V trial aims at evaluating the predictive value of an “endocrine responsiveness score” (ERS) [30]. This latter relies on the evaluation of the expression of ER and HER2 on CTC by using the CellSearch System. The ERS will be calculated based on the level of expression for these markers and the proportion of CTC showing such marker expression [30].

There are other ongoing large prospective interventional studies involving hormone-receptor positive MBC in which therapeutic decisions are based on CTC number and/or characterization [32]. Starting from the hypothesis that standard criteria for treatment decision between hormone therapy and chemotherapy may be weaker than CTC count, two trials have been designed. In the French STIC CTC trial (NCT01710605), it will be assessed the survival advantage of treating patients with a high CTC number with chemotherapy. COMETI P2 (NCT 01701050) is an American observational trial on ER positive, HER2 negative MBC patients that aims at determining a CTC-Endocrine Therapy Index to predict whether patients will respond favorably or not to a new endocrine therapy. The index will be defined by the evaluation on CTC, as assessed by CellSearch, of four biological markers: ER, Bcl2, HER2, and Ki67 [32].

### 3.3. Genomic Heterogeneity

Besides looking for known mutations, as described above, the development of whole genome amplification (WGA) followed by high-throughput sequencing (also called “next-generation sequencing”, NGS), microarray-based comparative genomic hybridization (array-CGH), and single-cell sequencing (SCS) techniques make it possible to profile single CTC [38,110]. Once optimized the best strategies to isolate single and pure CTC suitable for downstream analyses [48,111], this would allow to address important issues including: the ability of CTC to capture the genetic heterogeneity of primary and metastatic tumors (and therefore of all the possible drug targets); the variability of CTC isolated from the same patients; and the possibility, through CTC analyses, to monitor in real-time the appearance of resistant clones, possibly bearing druggable variants.

In fact, the initial studies were focused mainly on evaluating and comparing the presence of DNA mutations in CTC, primary tumors and metastases of the same patients. Heitzer’s group demonstrated by targeted gene sequencing that, in metastatic colon cancer, CTC presented most of the point mutations that were identified in the primary tumor [112]. Importantly, most mutations initially found only in CTC were subsequently de.tected at subclonal level in primary tumors and metastases, thus supporting the notion that CTC could provide an effective strategy to monitor tumor genomes [112]. In another study, single CTC, isolated from the blood of patients with lung adenocarcinoma, underwent exome sequencing and copy number profiling by using multiple annealing and looping-based amplification cycles (MALBAC) [113]. This study showed a quite strong concordance of the copy number profile in CTC, primary tumors and metastases, while point mutations highly differed [113]. Lohr’s group, instead, comparing in prostate cancer patients the exome of single sorted CTC with that of both metastases and different regions of the respective primary tumors, showed that the CTC profile could be already detected in a small clonal population of the primary tumor [114]. Moreover, it showed that CTC allowed capturing about half of the mutations present in primary tumors and metastases [114]. Interestingly, Dago’s group showed the prompt genomic evolution of CTC in a patient with a castrate-resistant prostate cancer undergoing chemotherapy treatment followed by a targeted therapy, suggesting that CTC analysis could monitor, in real-time, the disease evolution in treated patients [115]. In the case of MBC, a pilot study focused on the molecular characterization by NGS of 50 cancer-related genes of single CTC (detected by CellSearch and sorted by DEPArray), showed inter- and intra-patient heterogeneity in the mutational status of CTC, as well as discordance between the mutational status of the primary tumor and CTC [45]. Moreover, in one patient, the mutational profile of CTC before and during treatment shared only few sequence variants, possibly indicating that clones bearing these variants were resistant to the administered therapy [45]. Similarly, by targeted NGS of ~2200 mutations in 50 cancer genes, Shaw’s group analyzed CTC, enumerated by CellSearch and sorted by DEPArray, of 5 MBC patients [116]. In this case results were compared with those obtained by sequencing matched cfDNA and primary tumor tissue. Again, NGS analyses showed variability, among single CTC, in the mutational status of *PIK3CA*, *TP53*, *ESR1* and *KRAS* genes. Noteworthy, mutations of *ESR1* and *KRAS* genes were not detected in primary tumor tissues. This could either reflect the origin of CTC from a small clonal sub-population, already present in the primary tumor, or could be the result of the acquisition of a new mutation during disease progression [116]. Importantly, the mutations assessed in single CTC were also found analyzing the matched cfDNA, suggesting the possibility to analyze cfDNA in patients with low/negative CTC count [116]. *ESR1* mutations are acquired in patients who have received aromatase inhibitors and can determine a ligand-independent, constitutive ER activity. However, the level of resistance could differ depending on the specific mutations and on the drugs used [116]. Indeed, it has been recently reported that, in patients that progressed after the administration of aromatase inhibitors, the evaluation of the mutational status of *ESR1* in plasma samples could direct the choice of subsequent endocrine-based strategies [117].

Altogether these studies indicate that CTC sequencing methods could be useful to investigate spatial and temporal tumor heterogeneity and to provide a tool for a personalized medicine approach. However, a number of technical challenges, such as improving coverage uniformity, reducing technical error rates, dropping costs, enhancing throughput and developing new computational tools for analyzing large-scale SCS data sets, still lie ahead before they can be widely adopted by researchers and clinicians [110].

### 3.4. The Epithelial-to-Mesenchymal Transition as a Common Trigger of Different Types of Tumor Heterogeneity

According to recent findings, more invasive CTC may lose their epithelial antigens by an EMT process [74]. In fact, upon EMT epithelial cells acquire enhanced motility, invasiveness, apoptosis resistance, and the capability to modify the extracellular matrix [118]. The beginning and the completion of the EMT process requires the activation of multiple distinct molecular events, such as activation of transcription factors (e.g., twist, snail, slug, and forkhead box protein C2 (FOXC2)), expression of specific cell-surface proteins (e.g., N-cadherin), reorganization and expression of cytoskeletal proteins (e.g., increased vimentin expression, reduced cytokeratin expression), production of enzymes able to degrade the extracellular matrix, and modification in the level of specific microRNA (e.g., reduction in miR200 and increase in miR21 and miR10b) [118].

Upon Kalluri, 3 types of EMT can be recognized [118]. While type 1 EMT characterizes embryonic development and organogenesis, type 2 EMT plays a role in wound healing and fibrosis. Finally, type 3 EMT is associated with the invasive and metastatic behavior of the tumor. This latter form occurs in tumor cells to generate effects that are clearly different from those observed in type 1 and type 2 EMT (i.e., invasion and metastatization) by means of mechanisms that involve genetic and epigenetic changes of oncogenes and tumor suppressors. Notably, cancer cells may present EMT features at different extent: while some epithelial cells acquire only some mesenchymal traits, others become fully mesenchymal, losing all epithelial characteristics [118].

Signal transduction pathways that include transforming growth factor-beta 1 (TGF-β 1), rat sarcoma subfamily (RAS), as well as other growth factor and morphogen receptors (e.g., Notch, Wnt, and Hedgehog) may activate type 3 EMT [75,118]. Additionally, this process may be induced by transcription factors that include SNAIL1 and TWIST1 [75,118]. Importantly, by silencing the expression of the latter transcription factor, it is possible to reduce the frequency of CTC and the metastatic potential of a highly aggressive murine mammary cell line [119].

**Figure 2:**
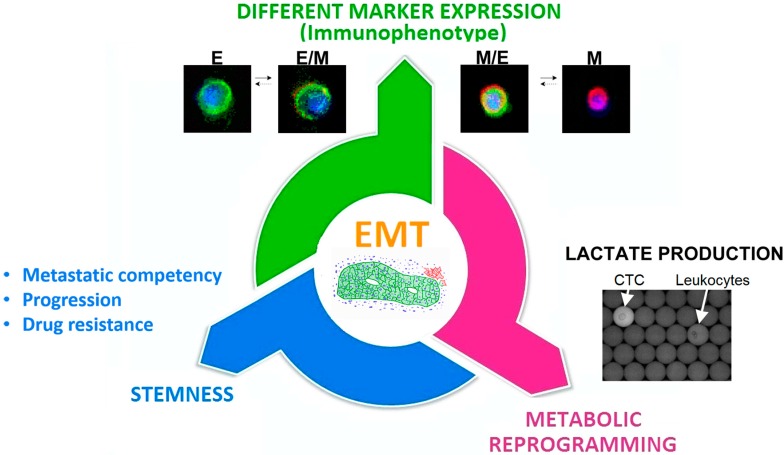
EMT can enhance tumor heterogeneity driving, in interested cells, phenotypic as well as functional changes increasing biological aggressiveness.

However, other key tumor features, such as stemness, drug resistance and changes in the metabolic properties, can be linked to the EMT process (Figure 2).

Regarding EMT and stemness, Mani in 2008 published a seminal paper that suggests a causal link between the two, in human mammary cell lines [84]. Similar results were shown by Morel et al. using a mammary tumor progression model [85]. Moreover, stem-like cells isolated from human normal and neoplastic tissues expressed mesenchymal markers [84]. Last, mammary cells that underwent EMT showed an aggressive biological behavior and formed tumors more efficiently when xenotransplanted in mice [84]. Accordingly, as previously described, EMT and stem cell markers are frequently coexpressed in CTC of breast cancer patients [42,54,55,76,77] and are related to poorer prognosis and drug resistance [120].

About this latter, EMT has been increasingly recognized as a key mechanism of cancer drug resistance [91]. In fact, numerous EMT-related signaling pathways are involved in drug resistance in cancer cells. Specifically, stem cell properties, which are associated with EMT and could increase the resistance of cells to toxic substances, are: the overexpression of multidrug resistance proteins and the resistance to apoptotic signaling [91]. As a consequence, it is conceivable that, by reducing EMT, it is possible to revert drug resistance. Interestingly, pathways important in EMT, such as Wnt/β-catenin, Hedgehog and Notch, play also a key role in maintaining self-renewal properties of stem cells [121]. As mentioned above, the ability of drugs targeting EMT and stem cell pathways to revert drug resistance is now under investigation in several clinical trials [57,88,89,90,91,93,120].

Additionally, it has been demonstrated that EMT can also be associated to profound metabolic changes [122,123]. As cited above, modifications of the tumor cellular bioenergetics, called “metabolic reprogramming”, are considered a hallmark of cancer and are strictly related to malignant transformation, invasion, metastasis, and drug resistance [94,124]. Besides the most studied aerobic glycolysis, termed the Warburg effect, other recognized metabolic features, which confer to cancer cells resistance to hypoxia and nutrient deprivation, include the so called “reverse Warburg effect”, “metabolic symbiosis” and “addiction to glutamine metabolism” [124]. Regarding aerobic glycolysis, breast cancer cell lines induced to undergo EMT displayed, with respect to the parental populations, an enhanced aerobic glycolysis, as indicated by high glucose uptake and lactate production rates [122]. Hypoxia inducible factor (HIF) may be responsible for the connection between the metabolic status and the invasive phenotype of the tumor. In fact, HIF-1α activation by hypoxia reduces the expression of E-cadherin [125,126], while it induces the expression of both the *met* proto-oncogene and TWIST [127,128]. Therefore, HIF-1α activation could entail both the metabolic reprogramming and the increased invasiveness and metastatization capacity of tumor cells. Importantly, CSC, with respect to the differentiated counterpart, exhibited a further metabolic shift and a mitochondrial resetting with a more pronounced Warburg effect [129,130,131]. Moreover, metabolic changes were also characterizing drug resistance phenomena. For example, in malignant melanoma cells resistant to an inhibitor of mutant BRAF (V600E), the chemotherapy induced a metabolic reprogramming probably responsible for the enrichment in CSC frequently seen in minimal residual disease [124,132]. Regarding CTC, we showed the possibility to identify CTC in the blood of metastatic patients taking advantage of their increased ability to produce hydrogenions and lactate [96]. Recently, it was demonstrated that colon cancer derived CTC, that expressed CD100 (i.e., thrombopoietin receptor), showed a strong liver tropism as a consequence of their enhanced lysine catabolism [133] and were characterized by several stem cell related features, thus suggesting that CTC could resemble CSC during the process of metastasis [133].

Altogether these evidences support the notion that indeed CTC in EMT can present properties, such as stemness, metabolic adaptation, enhanced invasiveness, migration ability and drug resistance, that could be responsible for a future metastatic spread. Alternatively, CTC in EMT can be viewed as a “circulating” representative of the most aggressive clones present in the patient tumor. Therefore, targeting EMT, or some of its related features, such as metabolic reprogramming, is opening the way to new therapeutic strategies aimed at inhibiting one of the most powerful mechanisms of cancer growth and evolutive adaptation.

## 4. Summary and Conclusions

Several studies have undoubtedly demonstrated the prognostic and predictive value of assessing the number, and/or changes in the number, of CTC in patients with early and metastatic breast cancers. However, CTC technologies have not yet been adopted in the routinely clinical management of many healthcare systems because a clear demonstration of their clinical utility is still missing. Indeed, the phase III clinical trial SWOG S0500, based on the recognition of epithelial CTC by the FDA-approved Cell Search system, failed in demonstrating that early switching to an alternative therapy, upon lack of CTC reduction, can improve the prognosis of MBC patients at the first line of therapy. Waiting for the results of other phase III interventional clinical trials based on CTC enumeration to guide therapy in other clinical settings (e.g., French CirCé01 trial and STIC CTC trials), nowadays the main area of investigation is the characterization of CTC.

Indeed, accumulated evidences support the notion that CTC are a heterogeneous population consisting of cells with different phenotype, genomic landscape as well as properties. Regarding phenotype, the evidence that CTC can undergo EMT with loss of epithelial markers, has favored the development of multiple new technologies aimed at recognizing CTC independently from the epithelial antigen expression. However, lacking at the moment a gold-standard test to measure all CTC populations in the blood, it is unclear which is the relationship between CTC isolated by different methods, often relying on different principles. This requires that for each novel CTC test, scientists must: define the context of use, obtain an analytical and a clinical validation, and demonstrate their clinical utility. Nowadays, only for few of them a clinical validation has been reached (Table 1).

Considering the genomic landscape, comprehensive single cell sequencing strategies have confirmed that CTC, at least in some clinical settings, are able to capture tumor heterogeneity and can allow a real-time monitoring of the appearance of drug-resistant clones, opening the way to use CTC as a liquid biopsy. Although a number of technical challenges still lie ahead before single cells sequencing techniques can be widely adopted by researchers and clinicians, they represent an invaluable opportunity for a personalized approach. Conversely, the detection of known-drug related molecules on CTC (i.e., ER and HER2) is already under evaluation in clinical trials. For example, DETECT III, a phase III interventional trial, is based on assessing CTC expressing HER2 in HER2-negative MBC. HER2^+^ cells will be further evaluated for the presence of activating PIK3CA mutations, possibly responsible for drug resistance to anti-HER2 therapies. Similarly, the translational research project of the DETECT V trial will evaluate the predictive value of an “endocrine responsiveness score” based on the expression of ER and HER2 on CTC.

Interestingly, it was shown that CTC could express EMT markers, as well as stem cell markers, and that this correlated with prognosis and drug resistance. EMT is a process that can induce in cancer cells many of the functional properties responsible of tumor aggressiveness, such as invasiveness, stemness, metabolic reprogramming and drug-resistance. Moreover, the pathways of these phenomena are overlapping and can be druggable. Indeed, several trials are now evaluating the ability of drugs possibly interfering with EMT and stemness, such as everolimus, to revert the drug-resistance to commonly used drugs, such as tamoxifene, nonsteroidal aromatase inhibitor and trastuzumab.

Additionally, we have shown that CTC in EMT can predict OS of patients from the time of metastasis occurrence, while the trial SWOG S0500 has suggested that patients in which CTC are not reduced after chemotherapy may represent a group of patients resistant to common chemotherapy. It would be therefore interesting to genetically and transcriptionally characterize CTC in EMT in drug-resistant patients to determine a putative sensitivity to alternative drugs.

Several groups, including our, have also shown that specific CTC sub-populations could be associated with specific metastatic sites. These studies, if confirmed, further expand the possible use of CTC to predict the metastatization pattern, thus paving the way to better understand (and possibly prevent) the metastatic process.

In conclusion, dissecting CTC heterogeneity can help in understanding either the metastatic potential of every single CTC or the aggressiveness of the tumor clone from which they derive. This would allow providing the most appropriate and personalized treatment for each patient, timely. However, in addition to the technological hurdles that still need to be resolved, only clinical trials can ultimately evaluate the clinical usefulness of this effort to go far beyond “the needle in the haystack”.

## 5. Methods

To identify the scientific literature regarding the heterogeneity of CTC in metastatic breast cancer and its possible clinical relevance we reviewed the literature in the PubMed database. We focused on publications written in English and published until August 2016. As search terms, we used “circulating tumor cells”, “metastatic breast cancer”, “CTC heterogeneity”, “epithelial to mesenchymal transition”, “metabolic reprogramming”, “cancer stem cells”, “single cell sequencing”, “metanalysis”, “progression free survival”, “overall survival”, “HER2 and hormone receptors on CTC”, “targeted therapy”, “precision medicine”, and “randomized trials in MBC”. Due to the large number of cited papers, we were not able to cite all individual references. We apologize to all authors whose important publications are not cited.

## Figures and Tables

**Table 1:** Clinical relevance of specific CTC (circulating tumor cell) subsets.

Method for CTC Detection	Subset of CTC Identified	Clinical Relevance	References
CellSearch assay (Veridex)	EpCAM^+^/CK8^+^/CD45^−^	A number of CTC ≥5 cells/7.5 mL at baseline and at the first follow-up represented an independent negative prognostic factor for OS and PFS.	[8,28,29,33,34]
	EpCAM^+^/CK^+^/CD45^−^	An elevated CTC number before the second cycle of chemotherapy was an early predictive marker of poor PFS and OS.	[35]
	EpCAM^+^/CK^+^/CD45^−^/M30^−/+^	The presence of M30-negative CTC was associated with a decreased chance of survival in metastatic patients. Both a decrease in the total CTC number and an increase in the fraction of apoptotic CTC (M30-positive) represented a predictive marker.	[36]
	EpCAM^+^/CK^+^/CD45^−^/HER2^±^	Evidence that HER2-negative primary tumors could develop HER2-positive CTC during disease progression. The HER2 status of CTC could be a prognostic factor in MBC patients.	[30,37,38,39,40]
Adna Test Breast Cancer	MUCIN-1-EpCAM^+^/HER2^±^	HER2-positive CTC could be detected in HER2-negative primary tumors.	[41]
	MUCIN-1-EpCAM^+^/Twist1^±^/Akt2^±^/Pl3Kα^±^/ALDH1^±^	CTC expressing EMT or stem cell-like markers were associated with poor prognosis and drug resistance.	[42]
	MUCIN-1-EpCAM^+^/HER2^±^/ER^±^/PgR^±^	The molecular profiling of CTC could predict the risk of recurrence and drug resistance.	[43]
DEPArray (Silicon Biosystems)	EpCAM^+^/CK^+^/CD45^−^	The mutational analysis of the TP53 status of CTC showed the presence of heterogeneity between CTC and primary tumors. The presence of TP53 mutations, as assessed by next-generation sequencing performed on single-cell sorted CTC, could represent a negative prognostic factor.	[44,45]
	4 CD45-negative subsets: Epithelial-CTC: EpCAM-E-cadherin^+^ EM-CTC: EpCAM-E-cadherin^+^/CD44-CD146-N-cadherin^+^ Mesenchymal cells: CD44-CD146-N-cadherin^+^/EpCAM^−^/E-cadherin^−^ Negative cells: EpCAM^−^/E-cadherin^−^/CD44^−^/CD146^−^/N-cadherin^−^	The presence of CTC in EMT was associated with a poor prognosis. The study highlighted also a correlation between the clinicopathological features of patients and the different subsets of CTC identified.	[46]
	EpCAM^+^/CK^+^/CD45^−^	The presence of activating PIK3CA mutations in CTC could predict resistance to anti-HER2 therapies.	[47,48]
Fluorescence in situ hybridization (FISH)	EpCAM^+^/CK^+^/CD45^−^/HER2 amplification^±^	Evidence that HER2-negative primary tumors developed HER2-positive CTC during disease progression, opening the way to targeted therapies.	[49]
Dual-colorimetric RNA-in situ hybridization	E-CTC: CK5-CK7-CK8-CK18-CK9-EpCAM-E-cadherin^+^ M-CTC: FN1-N-cadherin-SERPINE1-PAI1^+^	The mesenchymal immunophenotype was associated with disease progression. Furthermore, CTC from patients with lobular breast cancers were predominantly epithelial-like, whereas those from the triple negative and HER2-positive subtypes were predominantly mesenchymal-like.	[50]
Fluorescence activated cell sorting (FACS)	EpCAM^−^/HER2^+^/EGFR^+^/Heparanase^+^/Notch1^+^	Identification, on CTC, of a signature suggestive of metastatic competency to the brain.	[7]
EPISPOT (Epithelial ImmunoSPOT) assay	CK19^+^/MUCIN-1^+^	CTC releasing CK19 (CK19-RC) were correlated to an unfavorable clinical outcome.	[51]
ISET (isolation by size of epithelial tumor cells)	Size/CK7^+^	Evidence that EpCAM-negative CTC could escape from the CellSearch analysis.	[52]
RT-qPCR	CK19 mRNA	CK19 mRNA-positive cells could be detected in both early-stage and metastatic breast cancer patients, suggesting the use of RT-qPCR for the continuous monitoring and quantification of circulating epithelial cells.	[53]
	EM-CTC: EpCAM^+^/CD45^−^/TWIST1^+^/SNAIL1^+^/ZEB1^+^ Cancer stem cell-like cells: EpCAM^+^/CD45^−^/ALDH^+^/CD133^+^	EM-CTC and cancer stem cell-like cells had a prognostic value in HER2-positive MBC patients treated with targeted therapies.	[54]

## Abbreviations

ALDH1	Aldehyde dehydrogenase 1
cfDNA	Circulating free DNA
CSC	Cancer Stem Cells
CTC	Circulating Tumor Cells
EMT	Epithelial-to-Mesenchymal Transition
ER	Estrogen Receptor
EpCAM	Epithelial Cell Adhesion Molecule
EGFR	Epidermal Growth Factor Receptor
HER2	Human epidermal growth factor receptor 2
MBC	Metastatic Breast Cancer
NGS	Next Generation Sequencing
OS	Overall Survival
PgR	Progesteron Receptor
PIK3	Phosphatidylinositol 3-kinase
PIK3CA	Catalytic subunit of PIK3
PFS	Progression Free Survival
SCS	Single Cell Sequencing
